# Validation of a diagnosis-agnostic symptom questionnaire for asthma and/or COPD

**DOI:** 10.1183/23120541.00828-2020

**Published:** 2021-02-01

**Authors:** Niklas Karlsson, Mark J. Atkinson, Hana Müllerová, Marianna Alacqua, Christina Keen, Rod Hughes, Christer Janson, Barry Make, David Price, Helen K. Reddel, NOVELTY study investigators

**Affiliations:** 1 BioPharmaceuticals Medical, AstraZeneca, Gothenburg, Sweden; 2 Evidera, Bethesda, MD, USA; 3 BioPharmaceuticals Medical, AstraZeneca, Cambridge, UK; 4 BioPharmaceuticals R&D, AstraZeneca, Gothenburg, Sweden; 5 Dept of Medical Sciences: Respiratory, Allergy and Sleep Research, Uppsala University, Uppsala, Sweden; 6 National Jewish Health and University of Colorado Denver, Denver, CO, USA; 7 Observational and Pragmatic Research Institute, Singapore, Singapore; 8 Centre of Academic Primary Care, Division of Applied Health Sciences, University of Aberdeen, Aberdeen, UK; 9 Woolcock Institute of Medical Research, University of Sydney, Sydney, Australia

## Abstract

**Background:**

The Respiratory Symptoms Questionnaire (RSQ) is a novel, four-item patient-reported diagnosis-agnostic tool designed to assess the frequency of respiratory symptoms and their impact on activity, without specifying a particular diagnosis. Our objective was to examine its validity in patients with asthma and/or chronic obstructive pulmonary disease (COPD).

**Methods:**

Baseline data were randomly sampled from patients who completed the RSQ in the NOVELTY study (ClinicalTrials.gov: NCT02760329). The total sample (n=1530) comprised three randomly selected samples (n=510 each) from each physician-assigned diagnostic group (asthma, asthma+COPD and COPD). The internal consistency and structural validity of the RSQ were evaluated using exploratory and confirmatory factor analyses; psychometric performance was observed using Classical Test Theory and Item Response Theory analyses.

**Results:**

For the total sample, the mean±sd RSQ score was 5.6±4.3 (range 0–16). Irrespective of diagnosis, the internal consistency of items was uniformly adequate (Cronbach's α=0.76–0.80). All items had high factor loadings and structural characteristics of the measure were invariant across groups. Using the total sample, RSQ items informatively covered the θ score range of –2.0 to 2.8, with discrimination coefficients for individual items being high to very high (1.7–2.6). Strong convergent correlations were observed between the RSQ and the St George's Respiratory Questionnaire (0.77, p<0.001).

**Conclusions:**

The RSQ is a valid, brief, patient-reported tool for assessing respiratory symptoms in patients across the whole spectrum of asthma and/or COPD, rather than using different questionnaires for each diagnosis. It can be used for monitoring respiratory symptoms in clinical practice, clinical trials and real-world studies.

## Abstract 2

**The Respiratory Symptoms Questionnaire is a new, short, diagnosis-agnostic measure of respiratory symptoms. Validation in the NOVELTY study indicates that it is suitable for assessing respiratory symptoms in patients with asthma and/or COPD.** https://bit.ly/36Nw7eU

## Introduction

Respiratory symptoms are common and troublesome across the spectrum of obstructive lung disease, and are integral to the definitions of asthma and chronic obstructive pulmonary disease (COPD) [1, 2]. The presence of respiratory symptoms among current or ex-smokers with preserved lung function may also indicate a greater risk of exacerbations than among those who are asymptomatic [3]. However, many questionnaires that assess the frequency, impact or control of respiratory symptoms are designed specifically for patients with a confirmed diagnosis of either asthma or COPD [1, 2]. Questionnaires such as the Asthma Control Questionnaire (ACQ) and Asthma Control Test (ACT) provide composite scores of asthma symptom control [4]; however, neither is applicable for use in other obstructive lung diseases due to the inclusion of questions specifically referencing patients’ asthma [5, 6]. Furthermore, for patients with COPD, there are no similar symptom control questionnaires.

Three general respiratory symptom domains (breathing, coughing and chest symptoms) have been identified as common to both asthma and COPD [7–9]. These have been confirmed in qualitative concept elicitation patient interviews as part of two projects to develop diagnosis-specific symptom diary measures for asthma and COPD: the Asthma Daily Symptoms Diary, with a corresponding diary for night symptoms, and the EXACT (EXAcerbations of Chronic obstructive pulmonary disease Tool) for Respiratory Symptoms (E-RS: COPD) diary [7–9]. Some questions about general respiratory symptoms are included in several health-related quality-of-life questionnaires validated for use in asthma (*e.g.* the Asthma Quality of Life Questionnaire [10, 11]) or COPD (*e.g.* the Clinical COPD Questionnaire [12] and COPD Assessment Test [13]), but only two health status questionnaires have been validated for use in both conditions: the Airways Questionnaire 20 (AQ20) [14, 15] and St George's Respiratory Questionnaire (SGRQ) [11, 16].

However, there is no brief questionnaire for assessment of respiratory symptoms across asthma and COPD. Given the well-recognised overlap between asthma and COPD [17], and the increasing interest in developing more specific classifications of obstructive lung disease [18], there is a need for brief, “diagnosis-agnostic” (*i.e.* non-disease-specific) measures of respiratory symptoms in patients with asthma and/or COPD, so that the same tool can be used regardless of patients’ diagnostic labels. A brief diagnosis-agnostic tool would also be useful in clinical practice, especially in primary care, for monitoring patients with asthma and/or COPD, or who have not yet received a specific diagnosis.

The aim of this article is to describe the development of the Respiratory Symptoms Questionnaire (RSQ), and to assess its psychometric validity in patients with diagnoses of asthma and/or COPD.

## Methods

### Development of the RSQ

The RSQ was developed as a simple tool for use in both primary and specialist care settings to assess the frequency of respiratory symptoms and their impact on the patient's activity, without requiring a specific diagnostic label. Like the ACQ and ACT, the content of the RSQ was conceptually based on clinical guidelines for assessment of symptoms of asthma and of COPD [1, 2], *i.e.* the frequency of symptoms, and the extent to which they impact on the patient's activity. Furthermore, key symptoms assessed by the RSQ reflect the three symptom domains that are common to both asthma and COPD (breathing, coughing and chest symptoms).

**FIGURE 1:**
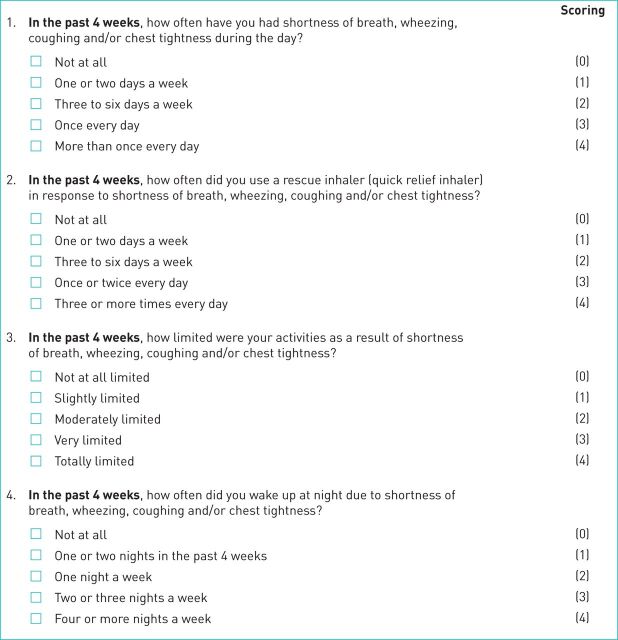
The Respiratory Symptoms Questionnaire (RSQ), response options and scoring. Responses to each of the four RSQ questions (items 1–4) are scored 0–4, with higher scores indicative of more frequent or worse symptoms. Total score ranges from 0 to 16. The RSQ is available for free use for legitimate scientific and clinical care reasons; AstraZeneca retains licensing rights through RWS Life Sciences (for further details, contact: astrazeneca@rws.com).

The RSQ comprises four questions (items 1–4) related to respiratory symptoms and their impact on the patient's activity: 1) frequency of symptoms of daytime shortness of breath, wheezing, coughing and/or chest tightness; 2) frequency of rescue (reliever) inhaler use in response to these symptoms; 3) degree of activity limitation due to these symptoms; and 4) frequency of night-time awakenings due to these symptoms (each over the previous 4 weeks) (figure 1). This is a similar structure to the Global Initiative for Asthma symptom control criteria [1]. RSQ score (range 0–16) is the sum score of the four items (scored 0–4), with higher scores indicative of worse symptoms. For valid scoring, all four items must be completed, otherwise RSQ scores are treated as missing.

Cognitive debriefing of RSQ items and response options was performed in patients with asthma (n=5) or COPD (n=5). RWS Life Sciences (East Hartford, CT, USA) employed a qualified recruiter to enlist potential patients in the USA and selected a convenience sample of patients who were native English speakers or literate and fluent in English. RWS Life Sciences selected a trained interviewer to conduct cognitive interviews; patients were guided through the RSQ and asked questions designed to capture their understanding and ensure that they could follow the instructions clearly. If patients expressed difficulty understanding, the interviewer asked questions to determine the reason. Overall, the patients were able to understand and use the RSQ; therefore, no changes were made to RSQ items or response options. Experts in linguistic validation from RWS Life Sciences provided harmonised translations of the original English questions into 19 languages.

### Psychometric validation of the RSQ

RSQ validation was conducted within a larger study: NOVELTY (a NOVEL observational longiTudinal studY; ClinicalTrials.gov: NCT02760329), which is a global, prospective, 3-year observational study of approximately 12 000 patients from 19 countries with a physician-assigned diagnosis or suspected diagnosis of asthma and/or COPD [19]. The aim of the present analysis was to use baseline data randomly sampled from NOVELTY to evaluate the reliability and validity of the RSQ as a measure of respiratory symptoms in patients with obstructive lung disease.

The NOVELTY study design has been reported previously [19]. Enrolment was stratified by physician-assigned diagnosis (asthma, both asthma and COPD (asthma+COPD) or COPD) and physician-assessed severity (mild, moderate or severe). No diagnostic or severity criteria were pre-specified when determining eligibility; diagnosis and assessment of severity were therefore decided by the physician. For patients with asthma+COPD, severity was allocated as the higher of the two severity categories assigned by the physician for their asthma and their COPD.

Patients in the NOVELTY study completed several patient-reported outcome (PRO) tools (by web-based platform or telephone) at the baseline visit or up to 21 days later [19]. Physicians were not aware of the responses to the RSQ or other PROs when assessing severity.

### Validation analysis design

This validation analysis used data obtained from NOVELTY patients’ responses to the RSQ, SGRQ, EuroQol Visual Analogue Scale (EQ VAS) and modified Medical Research Council (mMRC) dyspnoea scale (completed by all patients) and ACT (completed by patients with asthma or asthma+COPD).

Baseline data were randomly sampled from patients who had valid RSQ data (no missing responses) and had physician-assigned diagnoses of asthma, asthma+COPD or COPD. The total sample (n=1530) comprised three groups of 510 patients randomly selected from these diagnostic groups (asthma n=510 out of 3613, asthma+COPD n=510 out of 819 and COPD n=510 out of 2343). Sample sizes were selected to adequately power key psychometric analyses (supplementary material). Simple random sampling was expected to provide a balanced representation of severity categories (mild, moderate and severe) that was reflective of the NOVELTY study.

### Objectives

This analysis aimed to evaluate the reliability and validity of the RSQ as a diagnosis-agnostic (*i.e.* non-disease-specific) measure of respiratory symptoms in patients with physician-assigned asthma and/or COPD.

#### Primary objectives

The primary objectives were to: 1) assess the internal consistency, unidimensionality and structural validity of the RSQ to evaluate the coherence of the RSQ measure, and assess the structural and measurement invariance of the RSQ across diagnostic groups using confirmatory factor analysis; 2) examine the linearity of RSQ item response associated with physician-assessed severity categories using Classical Test Theory; 3) examine RSQ item information functions, to evaluate whether similar profiles were found across diagnostic groups and evaluate RSQ item response characteristics over the observed range of criterion metrics; and 4) examine the differential performance of RSQ items to sex and physician-assigned diagnosis using Item Response Theory, to determine the equivalence of RSQ item function for groups of interest.

#### Secondary objective

The secondary objective was to evaluate the construct validity of the RSQ. This included the convergent validity with measures expected to be related (*e.g.* SGRQ, EQ VAS, mMRC dyspnoea scale and physician-assessed severity) and the divergent validity with measures expected to be unrelated or weakly related (*e.g.* spirometry assessments). Additional analyses assessed the known-groups validity of the RSQ based on known-group characteristics (*e.g.* physician-assessed severity).

### Psychometric analyses

The psychometric performance of the RSQ was examined using two distinct statistical methods. The first set of methods examined the RSQ structural and measurement characteristics based on Classical Test Theory. Classical methods describe score distributions as being composed of both meaningful (true) score variance and random variation of scores (error). These analyses focused on the structural characteristics of the measure and the incremental difference in physician-assessed severity (mild, moderate or severe) observed at each RSQ response level. A second set of analyses was based on modern test theory (Item Response Theory) and used a graded response model to evaluate measurement performance of RSQ items based on the probability of endorsement of scaling options at different RSQ response thresholds along the severity/impact continuum. These analyses were performed using IRTPRO [20]. More detailed methodology for each analytical objective is provided in the supplementary material.

## Results

### Patient demographics and clinical characteristics

Patients with physician-assigned asthma were younger than those with asthma+COPD and COPD (mean age 52.7 *versus* 65.6 and 67.3 years, respectively) and a higher proportion were female (64.1% *versus* 45.1% and 38.4%, respectively) (table 1).

**TABLE 1:** Patient demographics and clinical assessments by physician-assigned diagnosis

	**Asthma**	**Asthma+COPD**	**COPD**	**Total sample**
**Patients**	510	510	510	1530
**Age years**	52.7±15.5	65.6±9.7	67.3±9.6	61.8±13.6
**Sex**				
Female	327 (64.1)	230 (45.1)	196 (38.4)	753 (49.2)
Male	183 (35.9)	280 (54.9)	314 (61.6)	777 (50.8)
**Ethnicity**				
African-American	22 (4.3)	19 (3.7)	21 (4.1)	62 (4.1)
Caucasian	371 (72.7)	395 (77.5)	424 (83.1)	1190 (77.8)
North East Asian	38 (7.5)	24 (4.7)	5 (1.0)	67 (4.4)
South East Asian	8 (1.6)	3 (0.6)	3 (0.6)	14 (0.9)
Other	71 (13.9)	69 (13.5)	57 (11.2)	197 (12.9)
**Time since diagnosis years**				
Patients with non-missing data	324 (63.5)	379 (74.3)	363 (71.2)	1066 (69.7)
Mean±sd	15.4±16.1	14.7±16.8	7.0±6.6	12.3±14.4
**Physician-assessed severity^#^**				
Mild	188 (36.9)	90 (17.6)	122 (23.9)	400 (26.1)
Moderate	186 (36.5)	223 (43.7)	150 (29.4)	559 (36.5)
Severe	136 (26.7)	197 (38.6)	238 (46.7)	571 (37.3)
**Post-BD FEV_1_ % pred**				
Patients with non-missing data	408 (80.0)	432 (84.7)	417 (81.8)	1257 (82.2)
Mean±sd	85.6±19.7	66.7±21.7	58.3±22.3	70.1±24.1
**SGRQ total score^¶^**				
Patients with non-missing data	507 (99.4)	508 (99.6)	507 (99.4)	1522 (99.5)
Mean±sd	29.8±21.0	40.2±22.4	41.9±22.0	37.3±22.4
**mMRC dyspnoea scale grade^+^**				
Patients with non-missing data	490 (96.1)	496 (97.3)	498 (97.6)	1484 (97.0)
Mean±sd	0.9±0.9	1.5±1.0	1.7±1.1	1.4±1.1
**ACT score^§^**				
Patients with non-missing data	495 (97.1)	454 (89.0)	NA	949 (62.0)
Mean±sd	19.6±4.5	17.6±5.1	NA	18.6±4.9
**RSQ score^ƒ^**				
Patients with non-missing data	510 (100.0)	510 (100.0)	510 (100.0)	1530 (100.0)
Mean±sd	4.4±3.8	6.4±4.4	6.0±4.3	5.6±4.3

Data are presented as n, mean±sd or n (%). COPD: chronic obstructive pulmonary disease; BD: bronchodilator; FEV_1_: forced expiratory volume in 1 s; SGRQ: St George's Respiratory Questionnaire; mMRC: modified Medical Research Council; ACT: Asthma Control Test; NA: not applicable; RSQ: Respiratory Symptoms Questionnaire. ^#^: for patients with asthma+COPD, severity was allocated as the higher of the two severity categories assigned by the physician for their asthma and their COPD; ^¶^: range 0–100; ^+^: range 0–4; ^§^: range: 5–25; ^ƒ^: valid scoring of the RSQ requires that all items have been completed and the sum score (range 0–16) is used (the total scores of individuals with missing values for any item were treated as missing and only patients with valid RSQ scores were randomly selected for this analysis).

More patients had mild asthma (36.9%) than mild COPD (23.9%) and more patients had severe COPD (46.7%) than severe asthma (26.7%) (supplementary table S1).

Patients with asthma had lower mean SGRQ scores *versus* those with asthma+COPD or COPD (table 1); these patterns were also observed across each severity category (supplementary table S1). Patients with asthma also had a higher mean post-bronchodilator forced expiratory volume in 1 s % predicted *versus* those with asthma+COPD or COPD overall (table 1) and across severity categories (supplementary table S1).

The mean±sd RSQ score in the total sample was 5.6±4.3 out of a maximum of 16, with lower scores among patients with asthma *versus* asthma+COPD or COPD (table 1).

### Structural validity

Differences in the dimensional structure of the RSQ between diagnostic groups were minimal and the internal consistency of items was uniformly adequate (Cronbach's α>0.7; asthma α=0.80, asthma+COPD α=0.79, COPD α=0.76 and total sample α=0.79). Additionally, all items had high factor loadings on exploratory factor analysis and thereby all contributed to the explained variance of a one-factor solution in a very similar way across all diagnostic groups (table 2). Using random samples, the structure of the RSQ and the measurement estimates it provided were invariant across diagnostic groups (supplementary table S2).

**TABLE 2:** Exploratory factor analysis using principal components factoring

	**Asthma**	**Asthma+COPD**	**COPD**	**Total sample**
**Patients**	510	510	510	1530
**Factor loadings for RSQ items**				
1^#^	0.800	0.819	0.798	0.812
2^¶^	0.760	0.761	0.734	0.758
3^+^	0.814	0.820	0.806	0.817
4^§^	0.817	0.757	0.744	0.770
**Eigenvalues^ƒ^**				
Factor 1	2.55	2.49	2.38	2.49
Factor 2	0.55	0.57	0.60	0.55
**Variance explained %**	63.7	62.4	59.4	62.4

Data are presented as n, unless otherwise stated. COPD: chronic obstructive pulmonary disease; RSQ: Respiratory Symptoms Questionnaire. ^#^: question 1 (“In the past 4 weeks, how often have you had shortness of breath, wheezing, coughing and/or chest tightness during the day?”); ^¶^: question 2 (“In the past 4 weeks, how often did you use a rescue inhaler (quick relief inhaler) in response to shortness of breath, wheezing, coughing and/or chest tightness?”); ^+^: question 3 (“In the past 4 weeks, how limited were your activities as a result of shortness of breath, wheezing, coughing and/or chest tightness?”); ^§^: question 4 (“In the past 4 weeks, how often did you wake up at night due to shortness of breath, wheezing, coughing and/or chest tightness?”); ^ƒ^: one factor was retained for each analysis based on a factor retention criteria of eigenvalue ≥1.00 (a second factor did not meet this threshold and there were no items with split loadings in a two-factor solution).

### Linearity of item response

**FIGURE 2:**
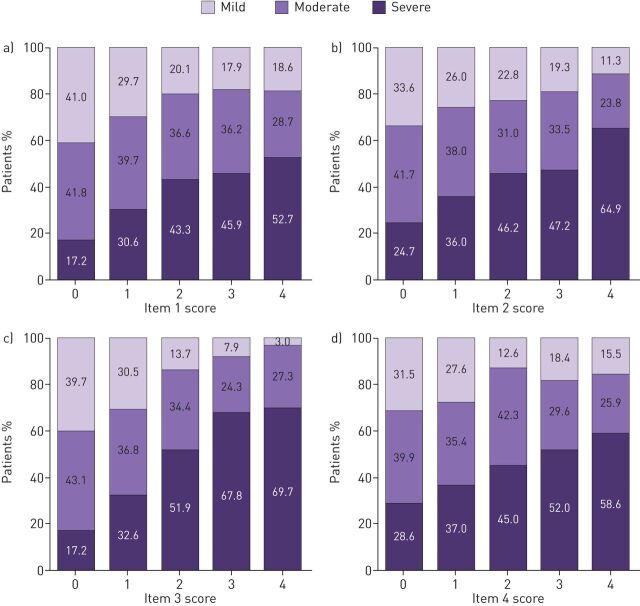
Distribution of physician-assessed severity (mild, moderate or severe) in the total sample for Respiratory Symptoms Questionnaire (RSQ) items, by response score: a) item 1 (frequency of daytime symptoms), b) item 2 (frequency of rescue inhaler use), c) item 3 (degree of activity limitation) and d) item 4 (frequency of night-time awakenings due to symptoms). The total sample (n=1530) comprised patients with physician-assigned diagnoses of asthma (n=510), asthma+COPD (n=510) and COPD (n=510). See figure 1 for full details of the RSQ questions and response options. Physicians were not aware of a patient's RSQ responses when they assigned the severity category. Percentages within columns may not total 100% due to rounding.

**FIGURE 3:**
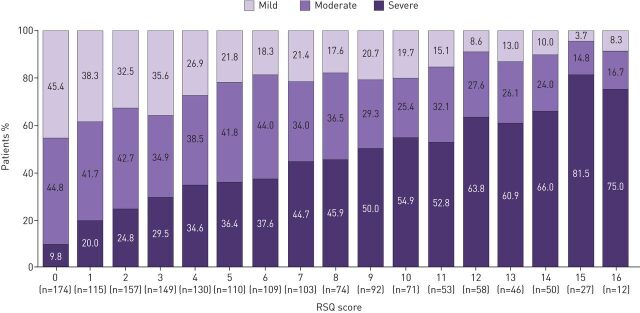
Distribution of physician-assessed severity (mild, moderate or severe) in the total sample, by Respiratory Symptoms Questionnaire (RSQ) score. The total sample (n=1530) comprised patients with physician-assigned diagnoses of asthma (n=510), asthma+COPD (n=510) and COPD (n=510). Percentages within columns may not total 100% due to rounding.

The response options for RSQ items were in the expected sequential order and all items exhibited linear relationships with physician-assessed severity, both in the total sample (figure 2) and in each physician-assigned diagnostic group (supplementary figure S1). There was also a linear relationship between RSQ total scores and severity categories in the total sample (figure 3). However, 9.8% (n=17 out of 174) of patients with an RSQ score of 0 for the previous 4 weeks had physician-assessed severity of “severe” (asthma n=4 out of 83, asthma+COPD n=5 out of 41 and COPD n=8 out of 50) and 8.3% (n=1 out of 12) of patients with an RSQ score of 16 had “mild” severity.

### Item response characteristics

Based on Item Response Theory methods, RSQ items informatively covered the difficulty Item Response Theory score range (θ). Here, “difficulty” refers to the location of a response on the symptom frequency and degree of activity limitation continuum (*i.e.* θ) as measured by the RSQ. Collectively across the total sample, θ lay between −2.0 and 2.8, and at least −1.6 to >2.8 for each physician-assigned diagnostic group, indicating that the RSQ provides similar information across diagnostic groups (supplementary figure S2). The discrimination coefficients for individual items were high to very high (range 1.7–2.6) and in line with items that provide a broad coverage of the response continuum (*i.e.* RSQ assessment dimension) [21]. The measurement precision estimates for the RSQ were adequate (>0.8), ranging from 0.79 to 0.81 [22].

### Differential item function

The absence of differential item functioning (a measure of the differences in the way items behave between discrete groups) was used to evaluate the equivalence of RSQ item function for groups of interest. No uniform differential item functioning was observed (supplementary table S3). Non-uniform differential item functioning was observed on one or two items between physician-assigned diagnostic groups (asthma *versus* COPD: items 3 and 4; asthma *versus* asthma+COPD: item 4; and COPD *versus* asthma+COPD: items 2 and 3) and on item 1 for sex (supplementary table S3).

### Construct validity

Convergent correlations between the RSQ and clinical variable assessments were highest for a health status measure (SGRQ) and ACT score (for patients with asthma), but were lower for a general health measure (EQ VAS), a single-symptom measure (mMRC dyspnoea scale) and physician-assessed severity (table 3). Divergent correlations were observed for spirometry measures (table 3).

**TABLE 3:** Convergent and divergent correlations between clinical assessments and total Respiratory Symptoms Questionnaire score

	**Asthma**	**Asthma+COPD**	**COPD**	**Total sample**
**Patients**	510	510	510	1530
**Convergent correlations**				
SGRQ total score	0.72***	0.77***	0.77***	0.77***
ACT score	−0.84***	−0.84***	NA	NA
EQ VAS	−0.43***	−0.54***	−0.51***	−0.52***
mMRC dyspnoea scale grade	0.41***	0.47***	0.44***	0.47***
Physician-assessed severity^#^	0.31***	0.33***	0.31***	0.34***
**Divergent correlations**				
Post-BD FEV_1_ % pred	−0.29***	−0.37***	−0.27***	−0.36***
Post-BD FVC % pred	−0.22***	−0.34***	−0.23***	−0.29***
Post-BD FEV_1_/FVC	−0.20***	−0.15**	−0.16**	−0.23***
Post-BD FEF_25–75%_ % pred	−0.18**	−0.07	0.00	−0.14***

Data are presented as n, unless otherwise stated. COPD: chronic obstructive pulmonary disease; SGRQ: St George's Respiratory Questionnaire; ACT: Asthma Control Test; NA: not applicable; EQ VAS: EuroQol Visual Analogue Scale; mMRC: modified Medical Research Council; BD: bronchodilator; FEV_1_: forced expiratory volume in 1 s; FVC: forced vital capacity; FEF_25–75%_: forced expiratory flow at 25–75% of FVC. For each variable, analyses were performed for patients with non-missing data; patient numbers differed for each variable based on data availability. Correlation coefficients >0.7 are regarded as strong, 0.4–0.7 as moderate and <0.4 as weak. ^#^: disease severity was categorised as mild, moderate or severe, as assigned by physician judgement (in order to calculate correlation coefficients, severity categories were assigned a numerical value: mild=1, moderate=2 and severe=3). **: p<0.01; ***: p<0.001.

### Known-groups validity

Table 4 presents RSQ scores by physician-assessed severity. Significance tests using standardised mean differences between groups showed that the RSQ differentiated all categories of severity within the total sample and the COPD group, as well as between mild or moderate *versus* severe categories within the asthma and asthma+COPD groups.

**TABLE 4:** Respiratory Symptoms Questionnaire (RSQ) differentiation of physician-assessed severity categories

	**Asthma**	**Asthma+COPD**	**COPD**	**Total sample**
**Patients**	510	510	510	1530
**RSQ scores^#^ by physician-assessed severity^¶^**				
Mild				
Patients with non-missing data	188	90	122	400
Mean±sd	3.32±3.27	5.06±4.13	4.11±3.71	3.95±3.67
Moderate				
Patients with non-missing data	186	223	150	559
Mean±sd	4.12±3.74	5.15±3.95	5.47±4.04	4.89±3.94
Severe				
Patients with non-missing data	136	197	238	571
Mean±sd	6.43±3.92	8.54±4.31	7.37±4.30	7.55±4.29
**RSQ differentiation by physician-assessed severity^¶^**				
Standardised mean differences^+^				
Mild *versus* moderate	0.23	0.02	0.35	0.25
Mild *versus* severe	0.87	0.82	0.79	0.89
Moderate *versus* severe	0.60	0.82	0.45	0.64
Group comparison				
Mild *versus* moderate	0.1042	0.9840	0.0249	0.0017
Mild *versus* severe	<0.0001	<0.0001	<0.0001	<0.0001
Moderate *versus* severe	<0.0001	<0.0001	<0.0001	<0.0001
F-value	30.02	41.46	27.61	109.85
p-value	<0.0001	<0.0001	<0.0001	<0.0001

Data are presented as n, unless otherwise stated. COPD: chronic obstructive pulmonary disease. ^#^: range 0–16; ^¶^: for patients with asthma+COPD, physician-assessed severity was allocated as the higher of the two severity categories assigned by the physician for their asthma and their COPD; ^+^: Cohen's d.

## Discussion

In this investigation of the psychometric properties of the RSQ (a simple, new respiratory symptoms questionnaire independent of diagnosis) the internal consistency and scale measurement structure of the tool were consistent across patients with physician-assigned diagnoses of asthma and/or COPD. The RSQ correlated well with the SGRQ, which, although a measure of health status in asthma and in COPD, includes multiple items addressing patient-reported respiratory symptoms and their impact. Strong correlations were also observed between the RSQ and ACT (for patients with asthma), which includes similar questions, also over 4 weeks. By comparison, weaker correlations were observed between the RSQ and mMRC dyspnoea scale (which assesses a single symptom, dyspnoea, without a defined time period), and with overall health status assessed by EQ VAS. Divergent correlations were observed with spirometry measures, consistent with other studies of respiratory symptom measures that have shown discordance between subjective and objective measures [23–26].

This evaluation was conducted among patients from the NOVELTY study [19], a large observational study that includes patients with physician-assigned diagnoses (or suspected diagnoses) of asthma, asthma+COPD and COPD [27]. Across all diagnoses, mean RSQ scores were greater with higher physician-assessed severity and statistically differentiated patients with severe disease from those with mild or moderate disease. Although differences in RSQ scores between mild and moderate disease in patients with asthma or asthma+COPD were not statistically significant, this was also apparent for other measures such as the ACT, SGRQ and mMRC dyspnoea scale; as such, it may reflect a lack of standardisation of severity assessment among physicians, as opposed to the performance of the RSQ. For example, although all RSQ items and RSQ total score exhibited an overall linear relationship with severity, ∼10% of patients with RSQ scores of 0 had a severity classification of “severe”. This suggests that respiratory symptoms may not be the predominant factor driving physicians’ severity assessments, and this may have contributed to the weak convergent correlations observed between the RSQ and physician-assessed severity. Physicians may have considered other factors such as comorbidities, treatment level (including biologic therapy) and exacerbation history. Currently, guidelines for assessing asthma severity advise identifying the patient's minimum effective treatment, which is not possible in an observational study [1], and there are no standardised recommendations for classifying COPD severity (except for severity of airflow limitation) [2]. Of note, when physicians classified severity they were not aware of patient responses to the RSQ or other PROs (*e.g.* ACT and SGRQ), with the exception of the mMRC dyspnoea scale grades, which were entered into electronic case report forms during visits.

Strengths of this analysis include the large real-world patient population recruited from active clinical practices in both primary and secondary care, the inclusion of patients with physician-assigned asthma, asthma+COPD and COPD, and the systematic recording of other relevant PROs. Within this broad context, the RSQ performed well using physician-assessed severity and various other criterion measures used in clinical practice. Limitations include that, like the ACT and ACQ, the content of the RSQ was conceptually based on clinical guidelines for assessing symptoms (primarily the extension of asthma symptom control criteria [1] to COPD [2] for the RSQ), so patient input into the development of the RSQ was limited to cognitive debriefing. Cognitive debriefing should be repeated for translated versions, given the variation in language about breathlessness between different ethnic groups [28]. An equal number of patients within each diagnostic label was randomly sampled from the NOVELTY population, so patients with asthma+COPD were relatively oversampled in this analysis (33%) *versus* the whole NOVELTY population (12%) [29]. This may affect the generalisability of results involving the total sample and asthma+COPD group. Future longitudinal data are required to further explore the performance of the RSQ, particularly to assess predictive validity, whether it reflects changes in respiratory symptoms over time and to provide estimates of a minimal clinically important difference.

Overall, the results of this analysis suggest that the RSQ is an effective tool for assessing respiratory symptoms in patients with asthma and/or COPD. Symptoms such as shortness of breath, wheeze, cough and chest tightness are characteristic of both asthma and COPD, and their diagnostic criteria overlap. There is substantial interest in developing more specific classifications of obstructive lung disease and in identifying underlying mechanisms, independent of conventional diagnostic labels [18, 19]. It is therefore essential to use the same tools for assessing respiratory symptoms across the whole spectrum of obstructive lung disease rather than using diagnosis-specific questionnaires for asthma and for COPD. Very few such tools are available. Both the ACT and E-RS: COPD diary focus on respiratory symptoms, but are diagnosis-specific. Questions about respiratory symptoms are included in several health status questionnaires such as the SGRQ and AQ20, but these tools are intended for assessing health status rather than only symptoms; they are also longer, *e.g.* the SGRQ takes 10–15 min to complete [16]. Unlike these tools, the RSQ is a diagnosis-agnostic tool that can assess respiratory symptoms consistently across the broad spectrum of obstructive lung disease. As there are only four questions, it can be completed in 1–2 min; this may be particularly valuable in primary care settings, where visit time is limited.

In conclusion, the results of this analysis demonstrate that the RSQ is a valid, easy-to-administer patient-reported tool for assessing and monitoring respiratory symptoms in patients with asthma and/or COPD. The RSQ is an effective diagnosis-agnostic measure that can be used for monitoring patient-reported respiratory symptoms in clinical practice, clinical trials and real-world studies. The RSQ may also be a valuable tool in studies to further our understanding of obstructive lung disease beyond conventional diagnostic labels or for patients in whom a diagnosis has not yet been made.

## Supplementary material

10.1183/23120541.00828-2020.Supp1 **Please note:** supplementary material is not edited by the Editorial Office, and is uploaded as it has been supplied by the author.
Supplementary material 00828-2020.SUPPLEMENT
Figure S1 00828-2020.FIGURES1
Figure S2 00828-2020.FIGURES2
Table S1 00828-2020.TABLES1
Table S2 00828-2020.TABLES2
Table S3 00828-2020.TABLES3
