# Supplementary material for: Validation of a diagnosis-agnostic symptom questionnaire for asthma and/or COPD
Source: ERJ Open Res. 2021 Feb 1;7(1):00828-2020. doi: 10.1183/23120541.00828-2020 (PMC7861031; doi:10.1183/23120541.00828-2020)
Supplement: Supplementary file 1 [file 00828-2020.SUPPLEMENT.pdf]

SUPPLEMENTARY MATERIAL

Sample size calculation

Sample sizes were selected to adequately power key psychometric analyses. The sample size required for Item Response Theory analyses was based on simulation studies that were similar to this validation analysis. The sample size selected (510 patients for each of the three diagnostic groups) was approximately double the minimum requirement proposed by Chen et al (250 patients) [1]. Furthermore, it has been found to provide adequate power (>80%) to detect differential item functioning for studies of this type [2].

Methodology for each analytical objective

Structural validity

The uni-dimensional structure of the RSQ was evaluated using exploratory factor analyses. A two-stage principal components factoring approach was used to first specify a solution with Eigen values >1 (resulting in a single factor), then specify an additional factor to evaluate the presence of a second factor. Cronbach's alpha scores were used to evaluate the internal consistency of patients' response patterns to RSQ items. Both analyses were performed for the total sample and for each physician-assigned diagnostic group.

Based on the findings of the exploratory factor analysis, a one-factor confirmatory factor analysis was pre-specified to examine the fit between a hypothetical single latent factor and the observed covariance structure. This allowed for examination of fit adequacy using model fit indices (*i.e.* chi-square, root mean square error of approximation and Bentler's comparative fit index). The invariance of the factorial and/or measurement characteristics of the RSQ was evaluated across physician-assigned diagnoses by comparing the same model parameters in each group. The confirmatory factor analyses used to demonstrate the factorial and measurement invariance of the RSQ were performed using Mplus v8.2.

Linearity of item response

Item responsiveness to physician-assessed severity was assessed by examining RSQ item and total response score distributions for each severity category and plotting results for the total sample and each physician-assigned diagnostic group.

Item response characteristics

The Item Response Theory parameters associated with RSQ items were examined to determine whether RSQ item and total test scores provided informative coverage of the hypothetical range of symptom frequency and degree of activity limitation (theta). The degree to which item rating scales discriminate between individuals scores on the theta continuum is the primary determinant of item and test information curves.

Differential item function

The absence of differential item functioning between discrete groups was used to evaluate the equivalence of RSQ item function for groups of interest. Differential RSQ item function was examined across discrete sex and diagnostic groups in the total sample: sex (male *versus* female); and physician-assigned diagnosis (asthma *versus* COPD, asthma *versus* asthma+COPD, and COPD *versus* asthma+COPD). These groups were evaluated for evidence of uniform and non-uniform differential item functioning using ordinal logistic regression (applied by DIFDETECT; a module for the Stata program [Stata v15.1]) and examination of item characteristic curves for RSQ items between differential item functioning samples [3, 4].

Construct validity

Pearson correlation coefficients were used to assess the convergent and divergent validity of the RSQ, with higher correlations indicative of similar performance. Measures assessed for correlation with the RSQ included physician-assessed severity, the SGRQ, the mMRC dyspnoea scale and the EQ VAS (for evaluation of convergent validity) and spirometry assessments (for evaluation of divergent validity). For patients with physician-assigned asthma or asthma+COPD, correlations between the RSQ and ACT were also assessed (for evaluation of convergent validity).

Known-groups validity

The known-groups validity of the RSQ was assessed by comparing results by physician-assessed severity using a SAS generalised linear model. Known-groups validity serves as a way to estimate the trans-temporal responsiveness of a measure using cross-sectional data; such analyses are also known as ‘responder analysis’. With this approach, groups known to differ on a characteristic of interest (in this case, physician-assessed severity) are compared to examine the difference between the results in the known groups (in this case, RSQ scores).

NOVELTY study investigators

The NOVELTY study investigators who have provided consent to be acknowledged in NOVELTY publications are listed in full below.

Argentina: Gabriel Benhabib, Xavier Bocca Ruiz, Ricardo del Olmo, Raul Eduardo Lisanti, Gustavo Marino, Walter Mattarucco, Juan Nogueira, Maria Parody, Pablo Pascale, Pablo Rodriguez, Damian Silva, Graciela Svetliza, Carlos F. Victorio, Roxana Willigs Rolon and Anahi Yañez.

Australia: Stuart Baines, Simon Bowler, Peter Bremner, Sheetal Bull, Patrick Carroll, Mariam Chaalan, Claude Farah, Gary Hammerschlag, Kerry Hancock, Zinta Harrington, Gregory Katsoulotos, Joshua Kim, David Langton, Donald Lee, Matthew Peters, Lakshman Prassad, Helen Reddel, Dimitar Sajkov, Francis Santiago, Frederick Graham Simpson, Sze Tai, Paul Thomas and Peter Wark.

Brazil: José Eduardo Delfini Cançado, Thúlio Cunha, Marina Lima, Alexandre Pinto Cardoso and Marcelo Rabahi.

Canada: Syed Anees, John Bertley, Alan Bell, Amarjit Cheema, Guy Chouinard, Michael Csanadi, Anil Dhar, Ripple Dhillon, J. Mark FitzGerald, David Kanawaty, Allan Kelly, William Killorn, Daniel Landry, Robert Luton, Piushkumar Mandhane, Andrew McIvor, Bonavuth Pek, Robert Petrella and Daniel Stollery.

China:[a] Meihua Chen, Yan Chen, Wei Gu, Kim Ming Christopher Hui, Manxiang Li, Shiyue Li, Ma Lijun, Guangyue Qin, Weidong Song, Wei Tan, Yijun Tang, Chen Wang, Tan Wang, Fuqiang Wen, Feng Wu, PingChao Xiang, Zuke Xiao, Shengdao Xiong, Jinghua Yang, Jingping Yang, Caiqing Zhang, Min Zhang, Ping Zhang, Wei Zhang, Xiaohe Zheng and Dan Zhu.

Colombia: Fabio Bolivar Grimaldos, Alejandra Cañas Arboleda, Carlos Matiz Bueno and Dora Molina de Salazar.

Denmark: Elisabeth Bendstrup, Ole Hilberg, Carsten Kjellerup and Ulla Weinreich.

France: Philippe Bonniaud, Olivier Brun, Pierre-Régis Burgel, Christos Chouaid, Francis Couturaud, Jacques de Blic, Didier Debieuvre, Dominique Delsart, Axelle Demaegdt, Pascal Demoly, Antoine Deschildre, Gilles Devouassoux, Carole Egron, Lionel Falchero, François Goupil, Romain Kessler, Pascal Le Roux, Pascal Mabire,

Guillaume Mahay, Stéphanie Martinez, Boris Melloni, Laurent Moreau, Chantal Raherison, Emilie Riviere, Pauline Roux-Claudé, Michel Soulier, Guillaume Vignal and Azzedine Yaici.

Germany: Sven Philip Aries, Robert Bals, Ekkehard Beck, Andreas Deimling, Jan Feimer, Vera Grimm-Sachs, Gesine Groth, Felix Herth, Gerhard Hoheisel, Frank Kanniess, Thomas Lienert, Silke Mronga, Jörg Reinhardt, Christian Schlenska, Christoph Stolpe, Ishak Teber, Hartmut Timmermann, Thomas Ulrich, Peter Velling, Sabina Wehgartner-Winkler, Juergen Welling and Ernst-Joachim Winkelmann.

Italy: Carlo Barbetta, Fulvio Braido, Vittorio Cardaci, Enrico Maria Clini, Maria Teresa Costantino, Giuseppina Cuttitta, Mario di Gioacchino, Alessandro Fois, Maria Pia Foschino-Barbaro, Enrico Gammeri, Riccardo Inchingolo, Federico Lavorini, Antonio Molino, Eleonora Nucera, Alberto Papi, Vincenzo Patella, Alberto Pesci, Fabio Ricciardolo, Paola Rogliani, Riccardo Sarzani, Carlo Vancheri and Rigoletta Vincenti.

Japan: Takeo Endo, Masaki Fujita, Takahiko Horiguchi, Keita Hosoi, Yumiko Ide, Minehiko Inomata, Hiromasa Inoue, Koji Inoue, Sumito Inoue, Motokazu Kato, Masayuki Kawasaki, Tomotaka Kawayama, Toshiyuki Kita, Kanako Kobayashi, Hiroshi Koto, Koichi Nishi, Junpei Saito, Yasuo Shimizu, Toshihiro Shirai, Naruhiko Sugihara, Ken-ichi Takahashi, Hiroyuki Tashimo, Keisuke Tomii, Takashi Yamada and Masaru Yanai.

Mexico: Ruth Cerino Javier, Alfredo Domínguez Peregrina, Marco Fernández Corzo, Efraín Montano Gonzalez, Alejandra Ramírez-Venegas and Adrian Rendon.

Netherlands: Willem Boersma, R.S. Djamin, Michiel Eijsvogel, Frits Franssen, Martijn Goosens, Lidwien Graat-Verboom, Johannes in 't Veen, Rob Janssen, Kim Kuppens, Maarten van den Berge and Mario van de Ven.

Norway: Ole Petter Brunstad, Gunnar Einvik, Kristian Jong Høines, Alamdar Khusrawi, and Torbjorn Oien.

South Korea: Yoon-Seok Chang, Young Joo Cho, Yong Il Hwang, Woo Jin Kim, Young-Il Koh, Byung-Jae Lee, Kwan-Ho Lee, Sang-Pyo Lee, Yong Chul Lee, Seong Yong Lim, Kyung Hun Min, Yeon-Mok Oh, Choon-Sik Park, Hae-Sim Park, Heung-Woo Park, Chin Kook Rhee, Ho Joo Yoon and Hyoung-Kyu Yoon.

Spain: Alvar Agustí García-Navarro, Rubén Andújar, Laura Anoro, María Buendía García, Paloma Campo Mozo, Sergio Campos, Francisco Casas Maldonado, Manuel Castilla Martínez, Carolina Cisneros Serrano, Lorena Comeche Casanova, Dolores Corbacho, Felix Del Campo Matías, Jose Echave-Sustaeta, Gloria

Francisco Corral, Pedro Gamboa Setién, Marta García Clemente, Ignacio García Núñez, Jose García Robaina, Mercedes García Salmones, Jose Maria Marín Trigo, Marta Nuñez Fernandez, Sara Nuñez Palomo, José Olaguibel Rivera, Luis Pérez de Llano, Ana Pueyo Bastida, Ana Rañó, José Rodríguez González-Moro, Albert Roger Reig and José Velasco Garrido.

Sweden: Dan Curiac, Christer Janson, Cornelia Lif-Tiberg, Anders Luts, Lennart Råhlen and Stefan Rustscheff.

UK: Frances Adams, Drew Bradman, Emma Broughton, John Cosgrove, Patrick Flood-Page, Liz Fuller, Timothy Harrison, David Hartley, Keith Hattotuwa, Gareth Jones, Keir Lewis, Lorcan McGarvey, Alyn Morice, Preeti Pandya, Manish Patel, Kay Roy, Ramamurthy Sathyamurthy, Swaminathan Thiagarajan, Alice Turner, Jorgen Vestbo, Wisia Wedzicha, Tom Wilkinson and Pete Wilson.

USA: Lo'Ay Al-Asadi, James Anholm, Frank Averill, Sandeep Bansal, Alan Baptist, Colin Campbell, Michael A. Campos, Bradley Chipps, Gretchen Crook, Samuel DeLeon, Alain Eid, Ellen Epstein, Stephen Fritz, Hoadley Harris, Mitzie Hewitt, Fernando Holguin, Golda Hudes, Richard Jackson, Alan Kaufman, David Kaufman, Ari Klapholz, Harshavardhan Krishna, Daria Lee, Robert Lin, Diego Maselli-Caceres, Vinay Mehta, James N. Moy, Ugo Nwokoro, Purvi Parikh, Sudhir Parikh, Frank Perrino, James Ruhlmann, Catherine Sassoon, Russell A. Settipane, Daniel Sousa, Peruvemba Sriram and Richard Wachs.

[a]Data for patients from China were excluded from the present analyses due to a change in regulations about data transfer in May 2019.

References

1. Chen WH, Lenderking W, Jin Y*, et al.* Is Rasch model analysis applicable in small sample size pilot studies for assessing item characteristics? An example using PROMIS pain behavior item bank data. *Qual Life Res* 2014; 23: 485-493.

2. Scott NW, Fayers PM, Aaronson NK*, et al.* A simulation study provided sample size guidance for differential item functioning (DIF) studies using short scales. *J Clin Epidemiol* 2009; 62: 288-295.

3. Gibbons L. DIFDETECT: Stata module to detect and adjust for differential item functioning (DIF). Statistical Software Components S458013. Boston College Department of Economics, 2015.

4. Gibbons L. RUNPARSCALE: Stata module to run PARSCALE from Stata. Statistical Software Components S456724. Boston College Department of Economics, 2006, revised 13 May 2015.
