# Supplementary material for: Validation of a diagnosis-agnostic symptom questionnaire for asthma and/or COPD
Source: ERJ Open Res. 2021 Feb 1;7(1):00828-2020. doi: 10.1183/23120541.00828-2020 (PMC7861031; doi:10.1183/23120541.00828-2020)
Supplement: Supplementary file 2 [file 00828-2020.FIGURES1.pdf]

SUPPLEMENTARY FIGURE S1 Distribution of response scores for a) RSQ item 1 (frequency of daytime symptoms); b) RSQ item 2 (frequency of rescue inhaler use); c) RSQ item 3 (degree of activity limitation); d) RSQ item 4 (frequency of night-time awakenings due to symptoms), by physician-assigned diagnosis and physician-assessed severity.

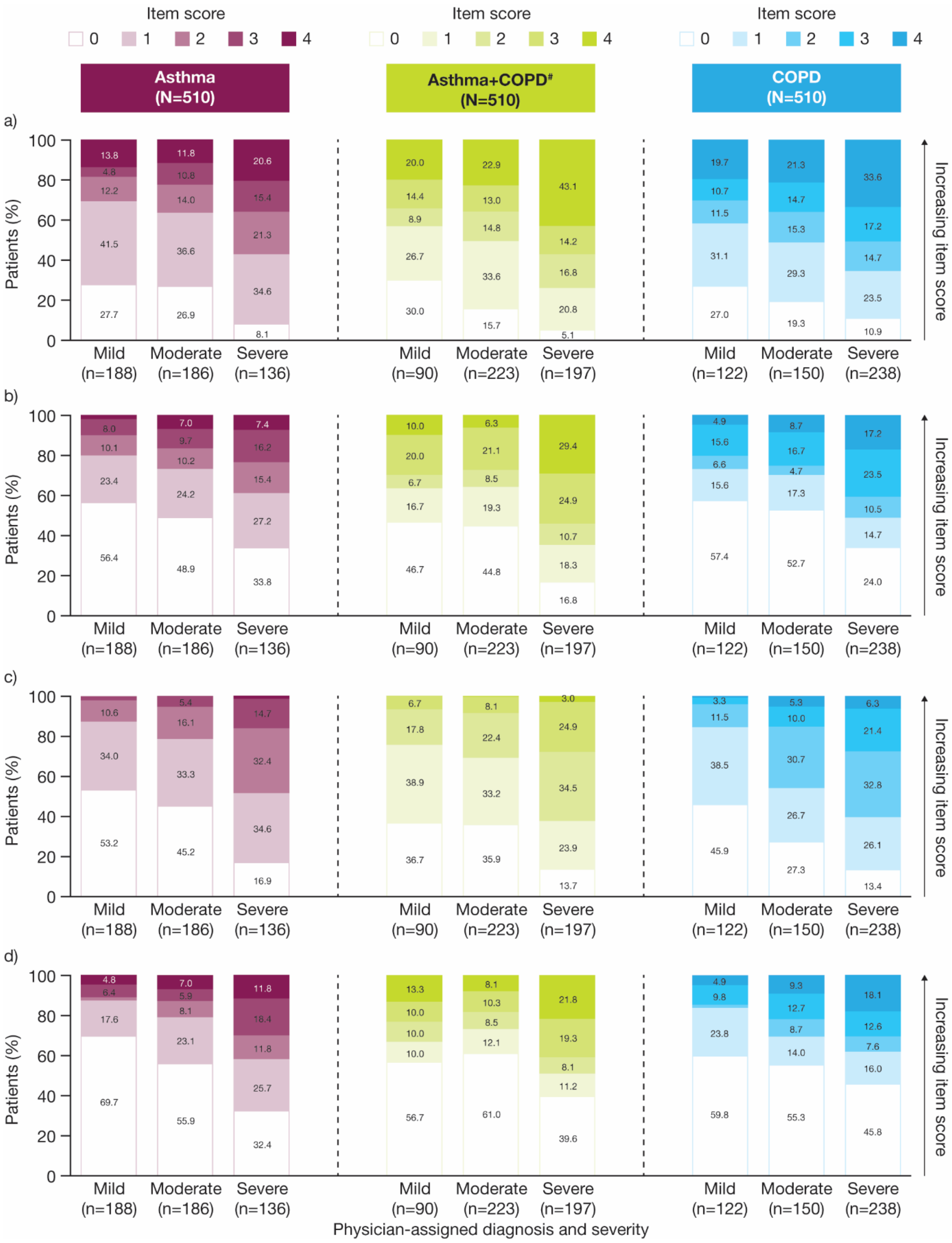

#: for patients with asthma+COPD, severity was allocated as the higher of the two severity categories assigned by the physician for their asthma and their COPD.
