# Supplementary material for: Validation of a diagnosis-agnostic symptom questionnaire for asthma and/or COPD
Source: ERJ Open Res. 2021 Feb 1;7(1):00828-2020. doi: 10.1183/23120541.00828-2020 (PMC7861031; doi:10.1183/23120541.00828-2020)
Supplement: Supplementary file 3 [file 00828-2020.FIGURES2.pdf]

**SUPPLEMENTARY FIGURE S2** Total test information functions by physician-assigned diagnosis and in the total sample.

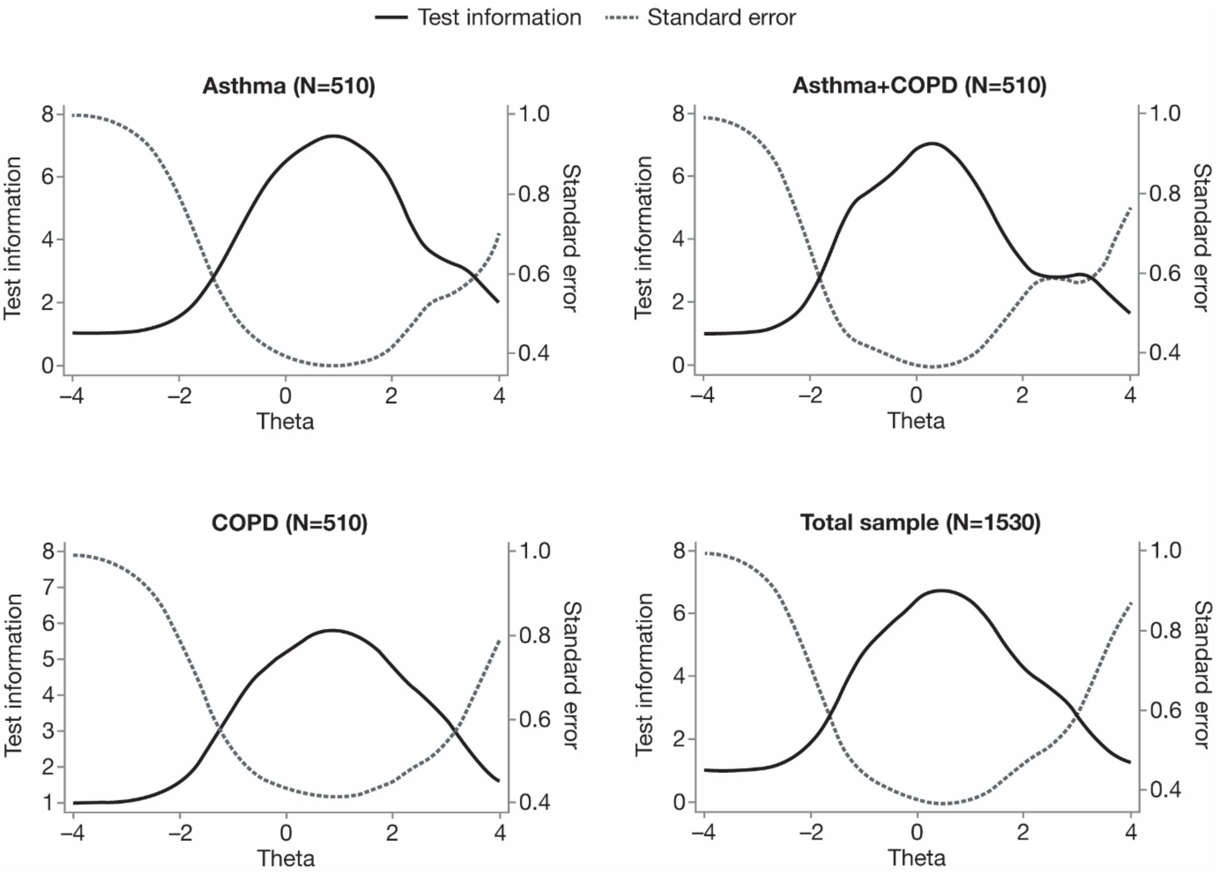


The total sample (N=1530) comprised patients with physician-assigned diagnoses of asthma (N=510), asthma+COPD (N=510) and COPD (N=510). Test information shows the level of information provided by the RSQ, over the difficulty Item Response Theory score range (theta). Here, theta is a continuum representing the hypothetical range of symptom frequency and degree of activity limitation as measured by the RSQ, and 'difficulty' refers to the location of a response on this continuum. Test information curves reflect the degree to which item rating scales discriminate between individuals scores on this continuum. The standard error shows the degree of error or imprecision in the RSQ estimate across the range of theta. Overall, the information function is the reciprocal of the estimation error and the locations; the RSQ measure describes the range of theta that occurs where the test information is greater than the standard error.
