# Supplementary material for: Validation of a diagnosis-agnostic symptom questionnaire for asthma and/or COPD
Source: ERJ Open Res. 2021 Feb 1;7(1):00828-2020. doi: 10.1183/23120541.00828-2020 (PMC7861031; doi:10.1183/23120541.00828-2020)
Supplement: Supplementary file 4 [file 00828-2020.TABLES1.pdf]

SUPPLEMENTARY TABLE S1 Patient demographics and clinical assessments by physician-assigned diagnosis and physician-assessed severity.

Variable	Asthma (N=510)			Asthma+COPD[#] (N=510)			COPD (N=510)		
	Mild (N=188; 36.9%)	Moderate (N=186; 36.5%)	Severe (N=136; 26.7%)	Mild (N=90; 17.6%)	Moderate (N=223; 43.7%)	Severe (N=197; 38.6%)	Mild (N=122; 23.9%)	Moderate (N=150; 29.4%)	Severe (N=238; 46.7%)
Age, years									
Mean (SD)	50.5 (16.1)	53.4 (15.9)	54.9 (13.9)	64.8 (10.4)	66.8 (9.4)	64.5 (9.6)	66.0 (10.4)	66.9 (10.7)	68.1 (8.3)
Sex, n (%)									
Female	129 (68.6)	115 (61.8)	83 (61.0)	43 (47.8)	103 (46.2)	84 (42.6)	51 (41.8)	60 (40.0)	85 (35.7)
Male	59 (31.4)	71 (38.2)	53 (39.0)	47 (52.2)	120 (53.8)	113 (57.4)	71 (58.2)	90 (60.0)	153 (64.3)
Ethnicity, n (%)									
African American	10 (5.3)	7 (3.8)	5 (3.7)	1 (1.1)	7 (3.1)	11 (5.6)	3 (2.5)	11 (7.3)	7 (2.9)
Caucasian	145 (77.1)	126 (67.7)	100 (73.5)	78 (86.7)	167 (74.9)	150 (76.1)	102 (83.6)	116 (77.3)	206 (86.6)
North East Asian	12 (6.4)	19 (10.2)	7 (5.1)	2 (2.2)	12 (5.4)	10 (5.1)	0 (0.0)	4 (2.7)	1 (0.4)
South East Asian	2 (1.1)	3 (1.6)	3 (2.2)	0 (0.0)	2 (0.9)	1 (0.5)	2 (1.6)	1 (0.7)	0 (0.0)
Other	19 (10.1)	31 (16.7)	21 (15.4)	9 (10.0)	35 (15.7)	25 (12.7)	15 (12.3)	18 (12.0)	24 (10.1)
Years since diagnosis									
Patients with data, n (%)	126 (67.0)	119 (64.0)	79 (58.1)	63 (70.0)	178 (79.8)	138 (70.1)	91 (74.6)	116 (77.3)	156 (65.6)
Mean (SD)	11.9 (15.1)	15.0 (15.7)	21.6 (16.6)	8.1 (11.6)	15.1 (16.7)	17.1 (18.1)	6.2 (7.8)	5.5 (5.1)	8.5 (6.5)
Post-bronchodilator FEV_1 % predicted									
Patients with data, n (%)	144 (76.6)	153 (82.3)	111 (81.6)	80 (88.9)	185 (83.0)	167 (84.8)	101 (82.8)	123 (82.0)	193 (81.1)
Mean (SD)	93.8 (16.4)	86.9 (17.0)	73.3 (21.2)	82.6 (15.6)	71.7 (17.5)	53.7 (21.2)	78.8 (19.8)	63.3 (16.6)	44.4 (16.4)
SGRQ total score[¶]									
Patients with data, n (%)	187 (99.5)	186 (100.0)	134 (98.5)	90 (100.0)	221 (99.1)	197 (100.0)	122 (100.0)	149 (99.3)	236 (99.2)

Mean (SD)	21.8 (16.0)	26.7 (20.2)	45.2 (20.2)	31.2 (18.9)	34.4 (20.4)	50.8 (21.9)	30.5 (19.1)	37.6 (22.0)	50.5 (19.8)
mMRC dyspnoea scale grade^{+}									
Patients with data, n (%)	186 (98.9)	174 (93.5)	130 (95.6)	88 (97.8)	215 (96.4)	193 (98.0)	119 (97.5)	148 (98.7)	231 (97.1)
Mean (SD)	0.6 (0.7)	0.8 (0.8)	1.6 (1.1)	1.0 (0.8)	1.3 (1.0)	1.9 (1.1)	1.0 (0.8)	1.5 (1.0)	2.3 (1.1)
ACT score§									
Patients with data, n (%)	182 (96.8)	181 (97.3)	132 (97.1)	82 (91.1)	192 (86.1)	180 (91.4)	NA	NA	NA
Mean (SD)	21.0 (3.6)	20.2 (4.4)	16.8 (4.5)	19.0 (4.0)	19.3 (4.5)	15.2 (5.3)	NA	NA	NA
RSQ score^{ƒ}									
Patients with data, n (%)	188 (100.0)	186 (100.0)	136 (100.0)	90 (100.0)	223 (100.0)	197 (100.0)	122 (100.0)	150 (100.0)	238 (100.0)
Mean (SD)	3.3 (3.3)	4.1 (3.7)	6.4 (3.9)	5.1 (4.1)	5.2 (4.0)	8.5 (4.3)	4.1 (3.7)	5.5 (4.0)	7.4 (4.3)

ACT: asthma control test; COPD: chronic obstructive pulmonary disease; FEV_1: forced expiratory volume in 1 second; mMRC: modified Medical Research Council; N: total number of patients in the sample; n: number of patients with non-missing data; NA, not applicable; RSQ: Respiratory Symptoms Questionnaire; SD: standard deviation; SGRQ: St George's Respiratory Questionnaire. $^{\#}$: For patients with asthma+COPD, severity was allocated as the higher of the two severity categories assigned by the physician for their asthma and their COPD; ¶: range: 0–100; $^{+}$: range: 0–4; §: range: 5–25; ^{ƒ}: valid scoring of the RSQ requires that all items have been completed and the sum score (range: 0–16) is used – the total scores of individuals with missing values for any item were treated as missing, and only patients with valid RSQ scores were randomly selected for this analysis.
