# Supplementary material for: Validation of a diagnosis-agnostic symptom questionnaire for asthma and/or COPD
Source: ERJ Open Res. 2021 Feb 1;7(1):00828-2020. doi: 10.1183/23120541.00828-2020 (PMC7861031; doi:10.1183/23120541.00828-2020)
Supplement: Supplementary file 5 [file 00828-2020.TABLES2.pdf]

SUPPLEMENTARY TABLE S2 Confirmatory factor analysis summary of invariance model testing fit statistics.

Model	Model constraints	X^2	df	p-value	CFI	RMSEA	SRMR	BIC	AIC
**Asthma *versus* COPD (random sample [n=1020])**									
1.0: Pooled asthma and COPD	None	4.71	2	0.095	0.998	0.036	0.090	12691.1	12632.0
1.1: Asthma *versus* COPD	Structural invariance[#]	56.93	8	0.000	0.957	0.110	0.085	12645.3	12546.7
1.2: Asthma *versus* COPD	Measurement invariance[¶]	72.52	8	0.000	0.944	0.126	0.096	12660.9	12562.3
**Asthma *versus* asthma+COPD and COPD *versus* asthma+COPD (random sample)**									
2.0: Pooled asthma, asthma+COPD and COPD (n=1530)	None	14.32	2	0.001	0.993	0.063	0.013	19216.1	19152.1
2.1: Asthma *versus* asthma+COPD (n=1020)	Structural invariance[#]	49.63	8	0.000	0.967	0.101	0.072	12490.5	12391.9
2.1.1: Asthma *versus* asthma+COPD (n=1020)	Measurement invariance[¶]	82.27	8	0.000	0.940	0.135	0.109	12523.1	12424.6
2.2: COPD *versus* asthma+COPD (n=1020)	Structural invariance[#]	142.38	8	0.000	0.880	0.181	0.146	13352.3	13253.7
2.2.1: COPD *versus* asthma+COPD (n=1020)	Measurement invariance[¶]	28.62	8	0.000	0.982	0.071	0.030	13238.5	13140.0

AIC: Akaike Information Criterion; BIC: Bayesian Information Criterion; CFI: Bentler's comparative fit index; COPD: chronic obstructive pulmonary disease; df: degrees of freedom; RMSEA: root mean square error of approximation; SRMR: standardised root mean square residual; X^2: Chi-square. [#]: Fixed factor loadings; [¶]: Fixed factor loadings and means.
