# Supplementary material for: Validation of a diagnosis-agnostic symptom questionnaire for asthma and/or COPD
Source: ERJ Open Res. 2021 Feb 1;7(1):00828-2020. doi: 10.1183/23120541.00828-2020 (PMC7861031; doi:10.1183/23120541.00828-2020)
Supplement: Supplementary file 6 [file 00828-2020.TABLES3.pdf]

SUPPLEMENTARY TABLE S3 Differential item functioning RSQ items by physician-assigned diagnosis or sex.

	RSQ item	Uniform DIF		Non-uniform DIF		
		**Uniform DIF**	**p-value (DIF)**	**Non-uniform DIF**	**p-value (DIF)**	**Direction of non-uniformity**
**Asthma *versus* COPD**	1[#]	No	0.853	No	0.403	Mixed
	2[¶]	No	0.059	No	0.205	Mixed
	3[+]	No	0.658	**Yes**	0.000	+
	4[§]	No	0.421	**Yes**	0.000	-
**Asthma *versus* asthma+COPD**	1[#]	No	0.752	No	0.606	Mixed
	2[¶]	No	0.287	No	0.251	Mixed
	3[+]	No	0.663	No	0.098	Mixed
	4[§]	No	0.972	**Yes**	0.000	Mixed
**COPD *versus* asthma+COPD**	1[#]	No	0.988	No	0.815	Mixed
	2[¶]	No	0.345	**Yes**	0.035	Mixed
	3[+]	No	0.124	**Yes**	0.002	-
	4[§]	No	0.418	No	0.791	Mixed
**Sex DIF**	1[#]	No	0.596	**Yes**	0.013	NA
	2[¶]	No	0.940	No	0.132	NA
	3[+]	No	0.278	No	0.078	NA
	4[§]	No	0.893	No	0.604	NA

Uniform DIF occurs when the group effect is consistent across the response continuum (*e.g.* an item could indicate that females scored higher than males across the entire range). Non-uniform DIF occurs when the group effect differs in the upper or lower end of the response continuum (*e.g.* males could indicate a greater effect of moderate to severe symptoms than females) [1]. No uniform DIF was observed, whereas non-uniform DIF was observed on one or two items between physician-assigned diagnostic groups, and one item for sex. P-value (DIF) <0.05 was indicative of DIF. COPD: chronic obstructive pulmonary disease; DIF: differential item functioning; NA: not applicable; RSQ: Respiratory Symptoms Questionnaire. [#]: Question 1 - 'In the past 4 weeks, how often have you had shortness of breath, wheezing, coughing and/or chest tightness during the day?'; [¶]: Question 2 - 'In the past 4 weeks, how often did you use a rescue inhaler (quick relief inhaler) in response to shortness of breath, wheezing, coughing and/or chest tightness?'; [§]: Question 3 - 'In the past 4 weeks, how limited were your activities as a result of shortness of breath, wheezing, coughing and/or chest tightness?'; [§]: Question 4 - 'In the past 4 weeks, how often did you wake up at night due to shortness of breath, wheezing, coughing and/or chest tightness?'.

References

1. Crane PK, Gibbons LE, Jolley L, van Belle G. Differential item functioning analysis with ordinal logistic regression techniques. DIFdetect and difwithpar. *Med Care* 2006; 44: S115-123.
